# Statistical learning and probabilistic prediction in music cognition: mechanisms of stylistic enculturation

**DOI:** 10.1111/nyas.13654

**Published:** 2018-05-11

**Authors:** Marcus T. Pearce

**Affiliations:** ^1^ Cognitive Science Research Group, School of Electronic Engineering and Computer Science Queen Mary University of London London UK; ^2^ Centre for Music in the Brain Aarhus University Aarhus Denmark

**Keywords:** music perception, enculturation, statistical learning, probabilistic prediction, IDyOM

## Abstract

Music perception depends on internal psychological models derived through exposure to a musical culture. It is hypothesized that this musical enculturation depends on two cognitive processes: (1) statistical learning, in which listeners acquire internal cognitive models of statistical regularities present in the music to which they are exposed; and (2) probabilistic prediction based on these learned models that enables listeners to organize and process their mental representations of music. To corroborate these hypotheses, I review research that uses a computational model of probabilistic prediction based on statistical learning (the information dynamics of music (IDyOM) model) to simulate data from empirical studies of human listeners. The results show that a broad range of psychological processes involved in music perception—expectation, emotion, memory, similarity, segmentation, and meter—can be understood in terms of a single, underlying process of probabilistic prediction using learned statistical models. Furthermore, IDyOM simulations of listeners from different musical cultures demonstrate that statistical learning can plausibly predict causal effects of differential cultural exposure to musical styles, providing a quantitative model of cultural distance. Understanding the neural basis of musical enculturation will benefit from close coordination between empirical neuroimaging and computational modeling of underlying mechanisms, as outlined here.

## Graphical abstract

It is hypothesized that this musical enculturation depends on two cognitive processes: statistical learning and probabilistic prediction. Here, I review research using the information dynamics of music model of probabilistic prediction based on statistical learning to suggest a single process underlying a broad range of psychological processes involved in music perception.

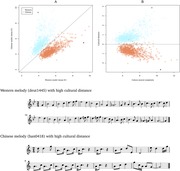

## Introduction

Musical styles comprise cultural constraints on the compositional choices made by composers, which can be distinguished both from constraints reflecting universal laws (of nature and human perception or production of sound) and specific within‐culture, nonstyle‐defining compositional strategies employed by particular (groups of) composers in particular circumstances.^1^ As recognized by Leonard Meyer in his early writing,^2^ these constraints can be viewed as complex, probabilistic grammars defining the syntax of a musical style,^3^, ^4^ which are acquired as internal cognitive models of the style by composers, performers, and listeners. This enables successful communication of musical meaning between composers and performers and between performers and listeners.^2^, ^5^, ^6^, ^7^, ^8^

Unlike many other general theories of music cognition,^9^, ^10^, ^11^, ^12^ this approach elegantly encompasses the idea that listeners exposed to different musical styles will differ in their psychological processing of music. It provides naturally for musical enculturation, the process by which listeners internalize the regularities and constraints defining and distinguishing musical styles and cultures. My purpose here is to elaborate Meyer's proposals by putting forward a computational model that is capable of learning the probabilistic structure of musical styles and examining whether the model successfully simulates the perception of mature, enculturated listeners across a broad range of cognitive processes and whether the model also simulates enculturation in musical styles.

I propose two hypotheses about the psychological and neural mechanisms involved in musical enculturation. According to these hypotheses, listeners use implicit statistical learning through passive exposure to acquire internal cognitive models of the regularities defining the syntax of a musical style; furthermore, they use probabilistic prediction based on the learned internal model to generate probabilistic predictions that underlie their perception and emotional experience of music. In other words, while existing theoretical approaches propose several distinct cognitive mechanisms underlying perception and emotional experience of music,^6^, ^9^, ^12^ here probabilistic prediction is put forward as a foundational mechanism underpinning other psychological processes in music perception. To substantiate these rather bold proposals, I introduce a computational model of probabilistic prediction based on statistical learning and present empirical results showing that the same model simulates a wide range of key cognitive processes in music perception (expectation, uncertainty, emotional experience, recognition memory, similarity perception, phrase‐boundary perception, and metrical inference). Finally, I demonstrate how the same model can be used to simulate enculturation and generate predictions about individual differences in perception resulting from enculturation in different musical styles.

## Statistical learning and predictive processing

Two hypotheses guide the present approach to understanding music cognition. The statistical learning hypothesis (SLH) states that musical enculturation is a process of implicit statistical learning in which listeners progressively acquire internal models of the statistical and structural regularities present in the musical styles to which they are exposed, over short (e.g., an individual piece of music) and long time scales (e.g., an entire lifetime of listening). The probabilistic prediction hypothesis (PPH) states that, while listening to new music, an enculturated listener applies models learned via the SLH to generate probabilistic predictions that enable them to organize and process their mental representations of the music and generate culturally appropriate responses.

*Probabilistic prediction* is the process by which the brain estimates the likelihood with which an event is likely to occur. With respect to musical listening, this corresponds to the probability of different possible continuations of the music (e.g., the next note or chord and its temporal position). But where do the probabilities come from? *Statistical learning* is the process by which individuals learn the statistical structure of the sensory environment and is thought to proceed automatically and implicitly.^13^, ^14^ This makes the theory general purpose in that it can potentially apply to any musical style, but also beyond music to other domains, such as language or visual perception. It also means that the theory can explicitly account for the effects of experience on music perception, including differences between listeners of different ages and different musical cultures and with different levels of musical training and stylistic exposure.

Research has established statistical learning and predictive processing as important mechanisms in many areas of cognitive science and cognitive neuroscience,^15^, ^16^, ^17^ including language processing,^13^, ^18^, ^19^, ^20^, ^21^ visual perception,^22^, ^23^, ^24^, ^25^ and motor sequencing.^26^ In particular, *predictive coding* 15, 17, 27, 28, 29 is a general theory of the neural and cognitive processes involved in perception, learning, and action. According to the theory, an internal model of the sensory environment compares top‐down predictions about the future with the actual events that transpire, and error signals generated from the comparison drive learning to improve future predictions by updating the model to reduce error. These prediction errors occur at a series of hierarchical levels, each reflecting an integration of information over successively larger temporal or spatial scales. Top‐down predictions are precision weighted such that more specific predictions (i.e., those more sharply focused on a single outcome) generate greater predictions errors. In the auditory modality, there is some evidence supporting hierarchical predictive coding for perception of nonmusical pitch sequences^30^, ^31^ and speech,^32^ though not all aspects of the theory have been empirically substantiated.^33^ Vuust and colleagues have proposed a predictive coding theory of rhythmic incongruity.^34^

As noted above, the idea that musical appreciation depends on probabilistic expectations has a venerable history, going back at least to Meyer's 1957 article.^2^ However, until relatively recently, empirical psychological research had been limited by the lack of a plausible computational model that simulates the psychological processes of statistical learning and probabilistic prediction. Recent research using the information dynamics of music (IDyOM) model^35^ has successfully implemented and extended Meyer's proposals and subjected them to empirical testing.

## IDyOM

IDyOM^35^ is a computational model of auditory cognition that uses statistical learning and probabilistic prediction to acquire and process internal representations of the probabilistic structure of a musical style. Given exposure to a corpus of music, IDyOM learns the syntactic structure present in the corpus in terms of sequential regularities determining the likelihood of a particular event appearing in a particular context (e.g., the pitch or timing of a note at a particular point in a melody). IDyOM is designed to capture several intuitions about human predictive processing of music.

First, expectations are dependent on knowledge acquired during long‐term exposure to a musical style,^36^, ^37^, ^38^ but listeners are also sensitive to repeated patterns within a piece of music.^39^, ^40^, ^41^ Therefore, IDyOM acquires probabilistic knowledge about a musical style through statistical learning from a large corpus reflecting a listener's long‐term exposure to a musical style (simulated by IDyOM's long‐term model (LTM), which is exposed to a large corpus of music in a given style). IDyOM also acquires knowledge about the structure of the music it is currently processing through short‐term incremental, dynamic statistical learning of repeated structure experienced during the current listening episode (simulated by IDyOM's short‐term model, which is emptied of any learned content before processing each new piece of music). Second, expectations are dependent on the preceding context, such that different expectations are generated when the context changes.^42^ In modeling terms, the length of the context used to make a prediction is called the *order* of the model. For example, a model that predicts continuations based on the preceding two events is a second‐order model (sometimes referred to as a trigram model). IDyOM is a variable‐order Markov model^43^, ^44^, ^45^, ^46^ that adaptively varies the order used for each context encountered during prediction. IDyOM also combines higher order predictions, which are structurally very specific to the context but may be statistically unreliable (because longer contexts appear less frequently, with fewer distinct continuations, in the prior experience of the model), with lower order predictions (based on shorter contexts) that are more structurally generic but also more statistically robust (since they have appeared more frequently with a wider range of continuations). IDyOM computes a weighted mixture of the predictions made by models of all orders lower than the adaptively selected order for the context.

**Figure 1:**
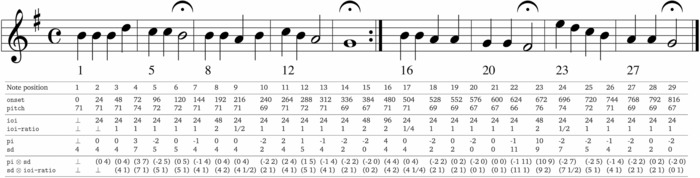
A chorale harmonized by J. S. Bach (BWV 379) showing examples of the input representations used by IDyOM. The first vertical panel shows the basic event space in which musical events are represented in terms of their chromatic pitch (pitch as an MIDI note number, where 60 = middle C) and onset time (onset, where 24 corresponds to a crotchet duration in this example). The second panel shows attributes derived from onset, including the interonset interval (ioi) and the ratio between successive interonset intervals (ioi‐ratio). Note that ioi is undefined (denoted by ⊥) for the first note in a melody, while ioi‐ratio is undefined for the first two notes. The third panel shows attributes derived from pitch, including the pitch interval in semitones formed between a note and its immediate predecessor (pi) and chromatic scale degree (sd) or distance in semitones from the tonic pitch (G or 67 in this example). The final panel shows two examples of linked attributes: first, linking pitch interval with scale degree (pi ⊗ sd) affording learning of combined melodic and tonal structure (the IDyOM models used Figs. 2, 3, 4 to use this linked attribute); second, linking pitch and temporal attributes (sd ⊗ ioi‐ratio), affording learning of combined tonal and rhythmic structure.

Third, research has demonstrated that listeners process music using multiple psychological representations of pitch^37^, ^47^, ^48^ (e.g., pitch height, pitch chroma, pitch interval, and pitch contour scale degree) and time^49^ (e.g., absolute duration‐based and relative beat‐based representations). Accordingly, IDyOM is able to create models for multiple attributes of the musical surface and combine the predictions made by these models. For example, it can be configured to predict pitch with a combination of two models for pitch interval and scale degree (see pi and sd in the third panel of Fig. 1). Alternatively, it can be configured to predict note onsets with a combination of two models for interonset interval and sequential interonset interval ratios (see ioi and ioi‐ratio in the second panel of Fig. 1).^35^, ^50^ Each of the models generates predictive distributions for a single property of the next note (e.g., pitch or onset time), which are combined separately for the long‐term and short‐term models before being combined into the final pitch distribution. Finally, listeners generate expectations for both the pitch^37^ and the timing of notes.^36^ Therefore, IDyOM applies the same process of probabilistic prediction described above in parallel to predict the pitch and onset time of the next note and computes the final probability of the note as the joint likelihood of its pitch and onset time. Given evidence that pitch structure and temporal structure are processed by listeners independently in some situations but interactively in others,^51^, ^52^, ^53^ IDyOM can process pitch and temporal attribute independently (using separate models whose probabilistic output is subsequently combined) or interactively using a single model of an attribute that links the two domains (e.g., by representing notes as a pair of scale degree and interonset interval ratio, see sd ⊗ ioi‐ratio in the lower panel of Fig. 1).

IDyOM acquires knowledge about the structure of music through statistical learning of variable‐length sequential dependencies between events in the music to which it is exposed and, while processing music event by event, generates expectations for the next event (e.g., the note that continues a melody) in the form of a probability distribution (*P*) that assigns a probability to each possible next event, conditioned upon the preceding musical context and the prior musical experience of the model. The information‐theoretic quantity *entropy* $\left(H=-\sum_{p\in P}p\log p\right)$ reflects the uncertainty of the prediction before the next event is heard—if every continuation is equiprobable, entropy will be maximum and the prediction highly uncertain, while if one continuation has very high probability, entropy will be low and the prediction very certain.^54^, ^55^ When the next event actually arrives, it may have a high probability, making it expected, or a low probability, making it unexpected. Rather than dealing with raw probabilities, *information content* ($h=-\log_{10}p$) provides a measure that is more numerically stable and has a meaningful information‐theoretic interpretation in terms of compressibility.^44^, ^54^ Information content (IC) reflects how unexpected the model finds an event in a particular context. Compression involves removing redundant information from a signal, which has been proposed as a central part of perceptual pattern recognition, and it has been argued that compression provides a measure of the strength of evidence for psychological interpretations of perceptual data (see also below).^56^, ^57^, ^58^

**Figure 2:**
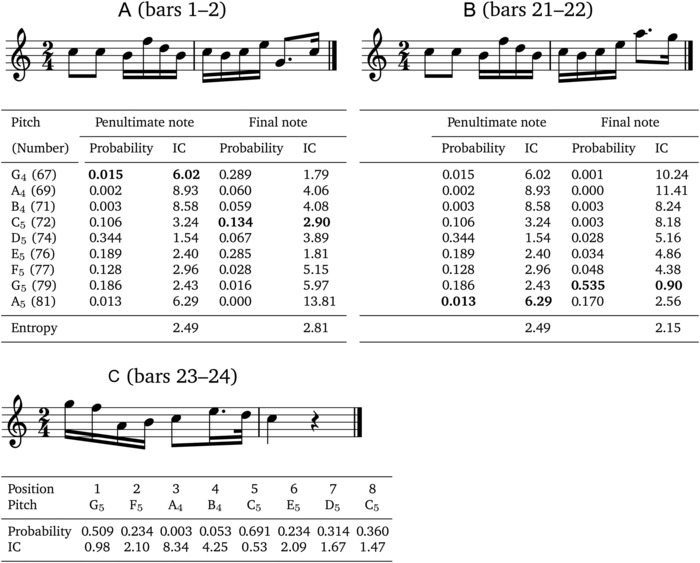
Three excerpts from the fourth movement of Schubert's *Octet in F Major* (D.803) taken from bars 1–2 (A), 21–22 (B), and 23–24 (C). (A and B) Probabilities and corresponding information content (IC) and entropy generated by IDyOM for the penultimate and final notes in each excerpt. At each point in processing, IDyOM estimates a probability distribution for the 37 chromatic pitches from B_2_ (47) to B_5_ (83), most of which have very low probabilities. For purposes of illustration, only the diatonic pitches between G_4_ and A_5_ are shown, including those that actually appear in the octet (highlighted in bold font). The entropy of the prediction is computed over the full 37‐pitch alphabet. (C) The probability and IC for each note appearing in the final two bars of the theme. In all cases, IDyOM was configured to predict pitch with an attribute linking melodic pitch interval and chromatic scale degree (pi ⊗ sd, see Fig. 1) using both the short‐term and long‐term models, the latter trained on 903 folk songs and chorales (data sets 1, 2, and 9 from table 4.1 in Ref. 35 comprising 50,867 notes).

Figure 2 applies IDyOM to excerpts from Schubert's *Octet for Strings and Winds*, which is discussed in detail by Leonard Meyer in his book *Explaining Music* (p. 219, example 121).^59^ Since Meyer's analysis pertains to pitch structure, IDyOM is configured only to predict pitch in this example. Referring to the penultimate note in the second bar (Fig. 2A), Meyer writes, “The continuation is triadic–to G–but in the wrong register. The realization therefore is only provisional.” IDyOM reflects this analysis, estimating a lower probability for the G_4_ that actually follows than for the G_5_ that is anticipated (0.015 versus 0.186). When the theme returns in bars 21–22 (Fig. 2B), Meyer writes that “The triadic implications of the motive are satisfactorily realized… But instead of the probable G, A follows—as part of the dominant of D minor (V/II).” IDyOM reflects this analysis, estimating a lower probability for the A_5_ that actually follows than for the G_5_ that is, again, anticipated (0.013 versus 0.186). The relatively high probability (0.344) assigned by IDyOM to the D_5_ can be attributed to another melodic process discussed by Meyer called *gap‐fill* in which a larger interval that spans more than one adjacent scale degree (the gap, C_5_–E_5_ in this case) creates an implication for the subsequent melodic movement to fill in the intervening scale degrees skipped over (here D_5_). The relatively high probability (0.189) assigned by IDyOM to the E_5_ reflects a general implication for small intervals (here a unison, the smallest interval possible).^10^ Meyer adds that “The poignancy of the A is the result not only of its deviant character and its harmonic context, but of the fact that the larger interval—a sixth rather than a fifth–acts both as a triadic continuation and as a gap implying descending motion toward closure.” Again, IDyOM reflects Meyer's analysis: the penultimate A_5_ in bar 22 allows IDyOM to predict the continuation with greater certainty than it could following the G_4_ in bar 2 (reflected in the lower entropy of 2.15 compared with 2.81), making the subsequent descent to the G_5_ (finally making its appearance, resolving the tension introduced by the preceding deviations from anticipated continuation) much more probable than it would have been following the penultimate G_4_ in bar 2 (0.535 versus 0.016) and indeed more probable than the C_5_ that actually followed in bar 2 (0.535 versus 0.134). As shown in Figure 2C, IDyOM also strongly anticipates the restatement of the G_5_ on the downbeat of bar 23, while the cadence toward tonal closure in the final two bars is characterized overall by high probability in IDyOM analysis (average probability = 0.3).

The features described above make IDyOM capable of simulating human cognitive processing of music to an extent that was simply not possible when Meyer was writing in the 1950s. Nonetheless, there are limits to the kinds of music (and musical structure) that IDyOM can process. To date, research has focused on modeling melodic music, generating predictions for the pitch and timing of individual notes based on the preceding melodic context (Figs. 1 and 2). However, recent research has extended IDyOM to modeling expectations for harmonic movement^60^ and has simulated melodic and harmonic expectations separately for tonal cadences in classical string quartets.^61^ Current research is also extending IDyOM to polyphonic music represented as parallel sequences, each containing a voice or perceptual stream, for which separate predictions are generated.^62^ In time, this approach may be capable of modeling complex aspects of polyphonic structure, such as stream segregation, and interactions between harmony and melody (e.g., the ways in which harmonic syntax constrains melodic expectations). IDyOM does require its musical input to be represented symbolically, which means that it cannot process aspects of music that rely on timbral, dynamic, or textual changes. Meyer refers to these parameters as secondary, since they do not usually take primary responsibility for bearing the syntax of a musical style (at least in the Western styles he is concerned with), and suggests that they operate differently from primary parameters (e.g., melody, harmony, and rhythm), though they may reinforce or diminish the effects of these syntactic parameters (which could be simulated as an independent process that is subsequently combined with IDyOM's predictive output). Where they take a prominent role in a musical style (e.g., electroacoustic music, electronic music, and soundscapes), I would predict that expectations are psychologically generated in a rather different way (based on extrapolation of physical properties, such as continuous changes in timbre, dynamics, or texture) that is not captured by IDyOM's structural processing of music.

Finally, it is instructive to draw parallels and contrasts between IDyOM and other modeling approaches, including rule‐based models, adaptive oscillator models, and general probabilistic theories of brain function. Rule‐based models have been proposed for simulating pitch expectations^10^, ^42^, ^63^, ^64^, ^65^ and temporal expectations.^9^, ^12^, ^66^, ^67^, ^68^ Such models are characterized by a collection of fixed rules for determining the onset and pitch of a musical event in a given context. Examples for pitch expectations are the *implication‐realization* theory^10^, ^63^ consisting of numerical rules defining the implications made by one pitch interval for the successive interval and the *tonal pitch space* theory^69^ consisting of numerical rules characterizing harmonic and melodic tension in terms of tonal stability and attraction. An example of a rule‐based approach to modeling temporal expectations is Melisma,^70^ which uses preference rules to select the preferred meter for a rhythm from a set of possible meters defined by well‐formedness rules. Rule‐based models depend heavily on the expertise of their designers and are often useful for analytical purposes, since the degree to which a musical example follows the rules can be interrogated perspicuously. However, since the rules are fixed and impervious to experience, such models cannot be used to simulate the acquisition of cognitive models of musical styles through enculturation (though they may describe the end result of this process for a given culture).

A rather different approach to simulating expectation is to use nonlinear dynamical systems, consisting of oscillators operating at different periods with specific phase and period relations.^71^, ^72^, ^73^, ^74^ In this approach, metrical expectations emerge from the resonance of coupled oscillators that entrain to temporal periodicities in the stimulus. A related oscillatory approach has been used to predict cross‐cultural invariances in perceived tonal stability.^75^ Since these models naturally imply an explanation of pitch and temporal processing in terms of stimulus structure, they do not provide a compelling account of enculturation (though it has been claimed that it is potentially compatible with Hebbian learning).^71^ It is possible that oscillator‐based models and the mechanisms of statistical learning and probabilistic processing implemented in IDyOM are complementary in simulating different aspects of expectation (e.g., enculturated versus nonenculturated processing) or by operating at different Marrian levels of description.^76^

More broadly, there are relationships between IDyOM and the general mechanisms of brain function hypothesized by predictive coding theory. First, although the representations in IDyOM input are particular to auditory stimuli, there is nothing else domain‐specific in IDyOM's design and, in fact, variable‐order Markov models are widely used in statistical language modeling^77^, ^78^ and universal lossless data compression.^44^, ^45^, ^46^ Second, IC is a measure of prediction error,^15^ as posited by predictive coding theory, between the event that actually follows and the top‐down prediction made by IDyOM based on prior learning: high IC implies greater prediction error and vice versa. Third, the combination of distributions produced by the subcomponent models within IDyOM is weighted by entropy such that models generating more certain predictions have higher weights.^35^, ^50^ This is similar to the precision weighting of prediction errors in predictive coding theory.^15^

## Probabilistic prediction in music cognition

To substantiate the proposal that probabilistic prediction constitutes a foundational process in music perception, the following sections review empirical results in which IDyOM models, after training on a corpus of Western tonal music, account well for the performance of Western participants (with long‐term exposure to Western tonal music) on a range of tasks, reflecting key psychological processes involved in music perception.

### Expectation and uncertainty

**Figure 3:**
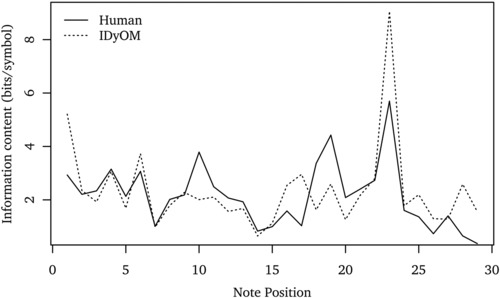
Information content generated by IDyOM for the Bach chorale shown in Figure 1, together with mean perceived expectedness from an empirical study reported by Manzara and colleagues.^84^ In this study, 15 participants were given a capital sum of virtual currency *S*_0_ = 0 and bet a proportion *p* of their capital on the pitch of each successive note in a melody (presented via a computer interface), continuing to place bets until the correct note was predicted, at which point they moved to the next note. At each note position *n*, incorrect predictions resulted in the loss of *p*, while the correct prediction was rewarded by incrementing the capital sum in proportion to the amount bet: $S_{n}=20pS_{n-1}$ (there were 20 pitches to choose from). The measure of information content plotted is derived by taking $\log_{2}20-\log_{2}S,$ where *S* is the capital won for a given note averaged across participants. As in Figure 2, IDyOM was configured to predict pitch with an attribute linking melodic pitch interval and chromatic scale degree (pi ⊗ sd, see Fig. 1) using both the short‐term and long‐term models, the latter trained on 903 folk songs and chorales (data sets 1, 2, and 9 in table 4.1 of Ref. 35 comprising 50,867 notes). IDyOM was configured to predict pitch only, since the participants in the Manzara *et al*. study were given the task of predicting pitch only.

IDyOM has been shown to predict accurately Western listeners’ melodic pitch expectations in behavioral, physiological, and electroencephalography (EEG) studies using a range of experimental designs, including the probe‐tone paradigm,^35^, ^79^ visually guided probe‐tone paradigm,^80^, ^81^ a gambling paradigm,^35^ continuous expectedness ratings,^82^, ^83^ and an implicit reaction‐time task to judgments of timbral change.^81^ In these studies, IC accounts for up to 83% of the variance in listeners’ pitch expectations. Furthermore, listeners show greater uncertainty when generating pitch expectations in low‐entropy contexts than they do in high‐entropy contexts, as predicted by IDyOM.^79^ In many circumstances, IDyOM provides a more accurate model of listeners’ pitch expectations than static rule‐based models,^10^, ^63^ which cannot account for enculturation.^35^, ^79^, ^80^ Figure 3 illustrates the relationship between IC and listeners’ expectations throughout a Bach chorale melody, using data from an empirical study of pitch expectations reported by Manzara *et al*.^84^

Furthermore, there is evidence that IC predicts neural measures of expectation violation. EEG studies with artificially constructed stimuli have identified an increased early negativity emerging around the latency of the auditory N1 (80–120 ms) for incongruent melodic endings in artificially composed stimuli.^85^, ^86^, ^87^, ^88^, ^89^, ^90^ Omigie *et al*. generalized these findings to more complex, real‐world musical stimuli, taking continuous EEG recordings while participants listened to a collection of isochronous English hymn melodies.^91^ The peak amplitude of the N1 component decreased significantly from high‐IC events through medium‐IC events to low‐IC events, and this effect was slightly right lateralized. Furthermore, across all notes in all 58 stimuli, the amplitude of the early negative potential correlated significantly with IC. Alongside the behavioral studies reviewed above,^35^, ^79^, ^80^, ^81^, ^82^, ^83^ these results show that IDyOM's IC also accounts well for neural markers of pitch expectation. It remains to be seen whether this holds true for neural measures of temporal expectation.^92^

### Emotional experience

Expectation is thought to be one of the principal psychological mechanisms by which music induces emotions.^6^, ^38^, ^93^, ^94^, ^95^ In spite of this, there has been very little empirical research that robustly links quantitative measures of expectation with induced emotion, partly due to the previous lack of a reliable computational model capable of simulating listeners’ musical expectations. Research has shown greater physiological arousal and subjective tension for Bach chorales manipulated to contain harmonic endings that violated principles of Western music theory^96^ and also for extracts from romantic and classical piano sonatas.^97^ However, as the stimulus categories were derived from music‐theoretic analysis, this does not provide insight into the underlying cognitive processes, especially with respect to the SLH and the PPH.

Egermann *et al*. took continuous ratings of subjective emotion (arousal and valence) and physiological measures (skin conductance and heart rate) while participants listened to live performances of music for solo flute. IDyOM was used to obtain pitch IC profiles reflecting the unexpectedness of the pitch of each note in the stimuli.^82^ The results showed that high‐IC passages were associated with higher subjective and physiological arousal and lower valence than low‐IC passages. This has been replicated in a controlled, laboratory‐based behavioral study of continuous responses to folk song melodies selected to vary systematically in terms of pitch and rhythmic predictability (assessed using IDyOM IC).^83^ The results showed that arousal was higher and valence lower for unpredictable compared with predictable melodies and that this effect was stronger for rhythmic predictability than pitch predictability. Furthermore, causal manipulations of the stimuli had the predicted effects on valence responses: transforming a melody to be more predictable resulted in increased valence ratings. Theoretical proposals of an inverted U‐shaped relationship between predictability and pleasure^98^ have received empirical support in some^99^ but not all^100^ studies of music perception. The results reviewed above show lower valence for more unpredictable musical passages, which may be because the particular combination of stimuli and participants reflects only the right‐hand side of a putative underlying an inverted U‐shaped relationship.

These results confirm the hypothesized role of probabilistic prediction in communicating musical affect, linking the predictability of musical events, assessed quantitatively in terms of IC, with the valence and arousal of listeners’ continuous emotional responses. Gingras *et al*. report a study that examines the relationship between compositional structure, expressive performance timing, and perceived tension in this communicative process.^8^ IDyOM was used to characterize, in terms of IC and entropy, the compositional structure of the *Prélude non mesuré No. 7* by Louis Couperin, which was then performed by 12 professional harpsichordists whose performances were rated continuously for tension experienced by 50 listeners. IC and entropy were predictive of continuous changes in performance timing (performers slowed down in anticipation of high‐IC events, and timing was more variable across performers around points of high IC and entropy), which, in turn, were predictive of perceived tension. Since the prelude is unmeasured, there is generous scope for expressive timing in performance, and, since the piece was performed on a harpsichord, performance expression is channeled primarily through timing, since there is little scope for expressive variations in dynamics and timbre. These design choices provide experimental control, but the results need to be generalized to a broader range of musical and instrumental styles.

It is important to note that expectation is not the only psychological mechanism by which music can induce emotions,^6^, ^93^ and future research should examine the ways in which expectation‐based induction of emotion interacts with other psychological mechanisms, such as imagery, contagion, and episodic memory, to generate complex aesthetic experiences of music.

### Recognition memory

As noted above, IDyOM uses computational techniques originally developed for use in universal lossless data compression, where IC has a well‐defined information‐theoretic interpretation.^44^, ^54^ A sequence with low IC is predictable and thus does not need to be encoded in full, since the predictable portion can be reconstructed with an appropriate predictive model; the sequence is compressible and can be stored efficiently. Conversely, an unpredictable sequence with high IC is less compressible and requires more memory for storage. Therefore, there are theoretical grounds for using IDyOM as a model of musical memory. Empirical research has shown that more complex musical examples are more difficult to hold in memory for later recognition,^101^, ^102^, ^103^, ^104^ and this appears to be related to features that are stylistically unusual.^105^ Furthermore, there is a strong link between information‐theoretic measures of predictability and perceived complexity of musical structure.^106^ Therefore, there are also empirical grounds for using IDyOM to simulate the relationship between stimulus predictability (as a measure of complexity) and memory for music.

Loui and Wessel used artificial auditory grammars to demonstrate that listeners show better recognition memory for previously experienced sequences generated by a grammar and that this generalizes to new exemplars from the grammar.^107^ Furthermore, in an EEG study, generalization performance correlated with the amplitude of an early anterior negativity (FCz, 150–210 milliseconds).^89^ However, this research did not explicitly relate degrees of predictability with memory performance. Agres *et al*. report a study that investigates recognition memory for artificial tone sequences varying systematically in information‐theoretic complexity across three sessions in each of which listeners were presented with 12 sequences, followed by a recognition test consisting of the same 12 sequences and 12 foils.^108^ To simulate listeners’ responses, an IDyOM model with no prior training was exposed to the stimulus set, learning the structure of the artificial style dynamically throughout the course of the session. In the first session, memory performance—measured by *d*′ scores—did not correlate with the average IC of the stimuli. However, over time, listeners learned the structure of the artificial musical style to the extent that, by the third session, IC accounted for 85% of the variance in memory performance, such that memory was better for predictable stimuli (those with low IC).

This suggests a strong relationship between the stylistic unpredictability of the stimulus, again represented by IDyOM IC, and accuracy of encoding or retrieval in memory. However, these results need to be replicated with actual music varying systematically in stylistic predictability.

### Perceptual similarity

Similarity perception is considered a fundamental process in cognitive science because it provides the psychological basis for classifying perceptual and cognitive phenomena into categories.^109^ Recent theories view the process of comparing two perceptual stimuli as a process of transformation such that similarity emerges as the complexity of the simplest transformation between them.^110^, ^111^, ^112^ This process can be simulated using information‐theoretic models as the *compression distance* between the two stimuli.^56^, ^113^, ^114^ Informally, IDyOM can be used to derive a compression distance *D(x, y)* between two musical stimuli *x* and *y* by training a model on *x*, using that model to predict *y*, and taking the average IC across all notes in *y* (see Ref. 115 for a formal presentation of the model). If *x* and *y* are very similar, the IC will be low; if they are very dissimilar, the IC will be high.

Pearce and Müllensiefen tested this model by comparing compression distance with pairwise similarity ratings provided by listeners in three studies for stimuli consisting of one original pop melody and a manipulated version (containing rhythm, interval, contour, phrase order, and modulation errors).^115^ The results showed very high correlations between compression distance and perceptual similarity (with coefficients ranging from 0.87 to 0.94), especially for IDyOM models configured to combine probabilistic predictions of pitch and timing.

To further assess generalization performance, IDyOM's measure of compression distance was tested on a very different set of data:^115^ the MIREX 2005 similarity task designed to evaluate melodic similarity algorithms in music information retrieval research.^116^, ^117^ In this task, algorithms must rank the similarity of 558 candidate melodies to each of 11 queries (all taken from the RISM A/II catalog of incipits from music manuscripts dated from 1600 onward), and performance is assessed by comparison with a canonical order compiled from the responses of 35 musical experts. Without any prior optimization for this task, IDyOM performed comparably to the best‐performing algorithms originally submitted (which took advantage of prior optimization on a comparable set of training data that is no longer available).

### Phrase‐boundary perception

The idea that perceptual grouping (or segment) boundaries occur at points of uncertainty or prediction error has been investigated in several areas of cognitive science, including modeling of phrase and word boundary perception in language.^118^, ^119^, ^120^ Research has also demonstrated that children and adults learn the statistical structure of novel artificial auditory sequences, identifying sequential grouping boundaries on the basis of low transition probabilities.^13^, ^121^

IDyOM has been used to test the hypothesis that perceived grouping boundaries in music (defining phrases) occur before contextually unpredictable events (those with high IC).^122^ The principle is illustrated clearly in Figure 3, in which phrase boundaries (marked by fermata in the score shown in Fig. 1) are preceded by a fall in IC to the final note of a phrase, followed by a marked rise in IC for the first note of the subsequent phrase. IDyOM was configured to predict both pitch and timing of notes and used to identify points where IC increased markedly compared with the recent trend.^122^ Comparing the boundaries predicted for 15 pop and folk songs with those indicated by 25 participants in an empirical study, IDyOM predicted perceived phrase boundaries with reasonable success. In most cases, performance was not as high as rule‐based models,^12^, ^123^ though these have been optimized specifically for phrase‐boundary detection based on expert knowledge and do not provide any account of enculturation or cross‐cultural differences in boundary perception.^124^ By contrast, IDyOM was not optimized in any way for boundary detection, and this research did not make full use of IDyOM's ability to simultaneously predict multiple attributes of musical events, leaving much scope for further development of IDyOM's phrase‐boundary detection model. Simulating boundary perception at one level opens the door to simulating perception of hierarchical structure in music by inferring embedded groups at different hierarchical levels of abstraction^11^ and using these as units in a multilayer predictive model.

### Metrical inference

The IDyOM models used to predict phrase‐boundary perception^122^ and similarity perception^115^ generate combined predictions of pitch and temporal position. In these models, the timing of notes is predicted using a model of statistical regularities in rhythm, but note timing is also influenced heavily by meter, a hierarchically embedded structure of periodically recurring accents that is inferred and aligned with a piece of music^9^ and is also an important influence on temporal expectations. Palmer and Krumhansl^36^ examined probe‐tone ratings for events whose timing was varied systematically in relation to the meter implied by the preceding rhythmic context. Ratings reflected the hierarchical structure of the meter and the statistical distribution of onsets in music, leading to the suggestion that listeners’ metrical expectations reflect learned temporal distributions.

Consistent with this proposal, cross‐cultural differences in meter perception have been observed using a task in which listeners detect changes to rhythmic patterns that either preserve or violate metrical structure.^125^, ^126^ American adults show better detection in *isochronous* meters (e.g., 6/8) than *nonisochronous* meters (e.g., 7/8), while adults from Turkey and the Balkans (where such meters are common) show no such difference^125^ but only for nonisochronous meters that appear in the culture.^127^ American 6‐month‐olds show no such difference in processing of isochronous and nonisochronous meters; 12‐month‐olds do show a difference, but it is eliminated by 2 weeks of listening to Balkan music, while this was not the case for U.S. adults.^126^ There is also evidence for cross‐cultural differences in rhythm production as a function of enculturation.^128^, ^129^

Can such enculturation effects be accurately simulated using computational models? As noted above, rule‐based models of meter perception^9^, ^12^, ^66^, ^67^, ^68^ are not sensitive to experience and therefore cannot plausibly account for enculturation, while approaches that simulate meter perception as emerging from the resonance of coupled oscillators that entrain to temporal periodicities^71^, ^73^, ^130^, ^131^ naturally imply an explanation of meter in terms of stimulus structure rather than the experience of the listener.

Recent research has extended IDyOM with an empirical Bayesian scheme for inferring meter.^132^ The metrical interpretation of a rhythm is treated as a hidden variable, consisting of both the metrical category itself (i.e., the time signature) and a phase aligning it to the rhythm. Metrical inference involves computing the posterior probability of a metrical interpretation at a given point in a rhythm through Bayesian combination of a prior distribution over meters (estimated empirically from a corpus) with the likelihood of an onset given the meter (estimated empirically by IDyOM). By virtue of IDyOM's statistical modeling framework, both the likelihood and the prior are also conditional on the preceding rhythmic context; therefore, metrical inference can vary dynamically event by event during online processing of music, taking into account the previous rhythmic context. Furthermore, the model naturally combines IDyOM's temporal predictions arising through repetition of rhythmic motifs with temporal predictions arising from the inferred meter. Unlike other probabilistic approaches, which are hand‐crafted specifically for meter finding,^133^, ^134^ this approach derives metrical inference from a general‐purpose model of sequential statistical learning and probabilistic prediction (implemented in IDyOM).

Computational simulations suggest that the model of metrical inference performs well. In a collection of 4966 German folk songs from the Essen Folk Song Collection,^135^, ^136^, ^137^ it correctly predicted the notated time signature in 71% of the corpus, with performance increasing for higher order models (tested up to an order bound of four). Furthermore, and of greater theoretical interest, metrical inference substantially reduces IC (or prediction error) at all order bounds compared with a comparable IDyOM model of temporal prediction that does not perform metrical inference. This provides concrete, quantitative evidence that metrical inference is a profitable strategy for improving accuracy of temporal prediction in processing music. It is important to generalize these findings to musical styles exhibiting a greater range of meters (including nonisochronous meters), as well as styles exhibiting high levels of metrical uncertainty (e.g., through syncopation or polyrhythm), making metrical induction more challenging.

## Statistical learning in musical enculturation

Most research on music cognition has been conducted on Western musical styles guided, implicitly or otherwise, by the particularities of Western music theory. However, the syntactic structure of musical styles varies among musical cultures. According to the SLH, this structure is learned through exposure producing observable differences among listeners from different musical cultures. Demorest and Morrison capture the effects of the SLH in their *cultural distance* hypothesis: “the degree to which the musics of any two cultures differ in the statistical patterns of pitch and rhythm will predict how well a person from one of the cultures can process the music of the other.”^138^ While cross‐cultural research has found evidence of differences in music perception between listeners as a function of their culture,^40^, ^41^, ^64^, ^65^, ^124^, ^125^, ^126^, ^127^, ^128^, ^129^, ^139^, ^140^, ^141^, ^142^, ^143^, ^144^, ^145^, ^146^ the psychological mechanisms underlying the acquisition of these differences are currently poorly understood.

The research reviewed to this point demonstrates that exactly the same underlying model of probabilistic prediction provides a plausible account of a wide range of different psychological processes in music perception, including expectation, emotion, recognition memory, similarity perception, phrase‐boundary perception, and metrical inference. In this research, the responses of Western listeners have been simulated using IDyOM models trained on Western tonal music (that approximates, within a tolerable degree of error, the stylistic properties of the music to which a typical Western listener is exposed). The IDyOM results reviewed above, therefore, are consistent with statistical learning as a mechanism for musical enculturation but the relationship is correlational rather than causal (with the exception of Ref. 108, which examined statistical learning directly but using an artificial musical system). In the following, I will outline a new modeling approach for a causal empirical investigation of the SLH of enculturation in musical styles.

**Figure 4:**
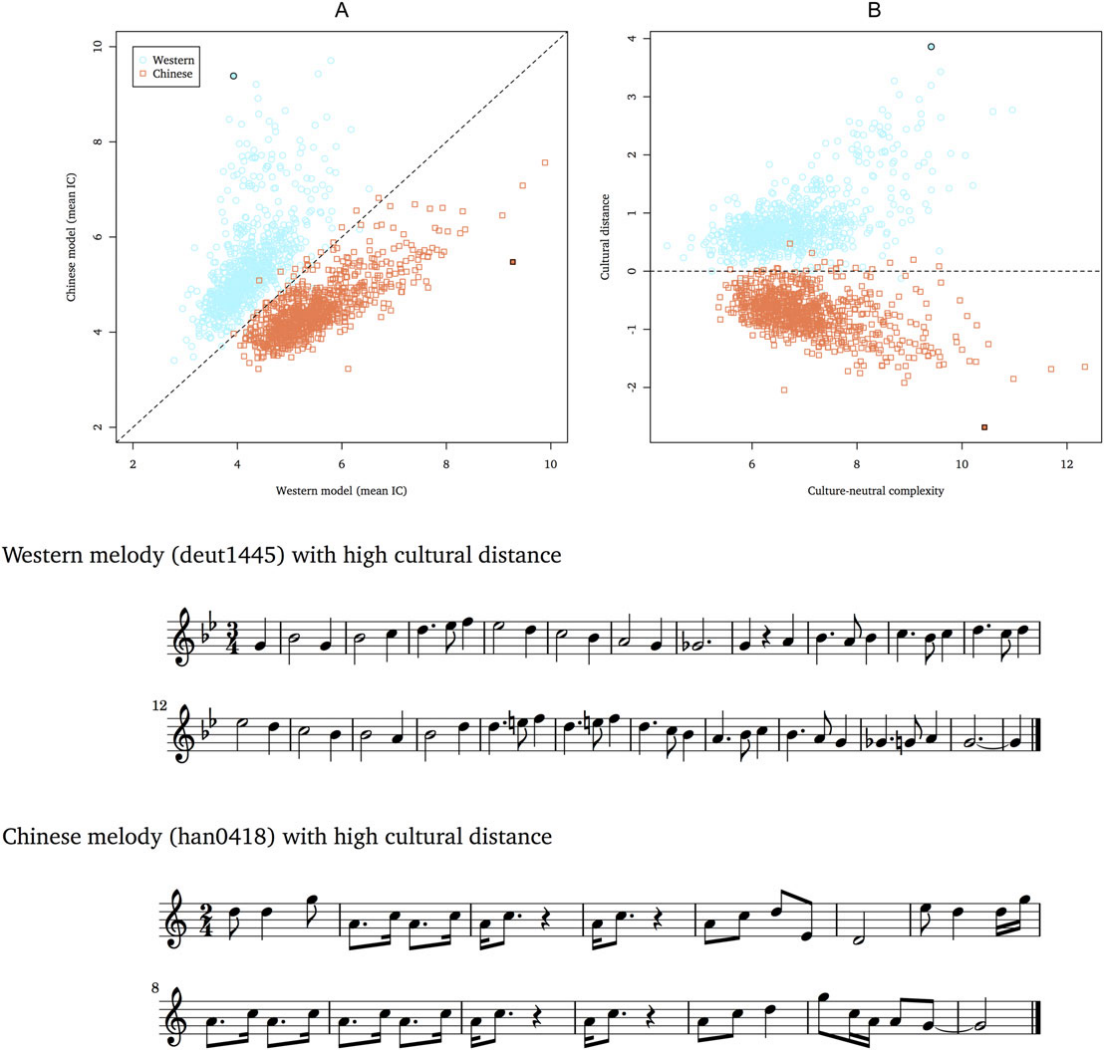
Simulating cultural distance between Western and Chinese listeners. (A) The information content of the Western model plotted against that of the Chinese model with the line of equality shown. (B) A 45° rotation of A such that the ordinate represents cultural distance and the abscissa culture‐neutral complexity. For each style, the composition with the most extreme cultural distance is highlighted, and corresponding musical scores are shown for these two melodies. The Western corpus consists of 769 German folk songs from the Essen Folk Song Collection^135^, ^136^, ^137^ (data sets *fink* and *erk*). The Chinese corpus consists of 858 Chinese folk songs from the Essen Folk Song Collection (data sets *han* and *natmin*). In a prior step, duplicate compositions were removed from the full data sets using a conservative procedure that considers two composition duplicates if they share the same opening for melodic pitch intervals, regardless of rhythm. IDyOM is configured to predict pitch with an attribute linking pitch interval with scale degree (pi ⊗ sd) and onset with the ioi‐ratio attribute (Fig. 1) using the long‐term model only trained on the Western and Chinese corpora, respectively, for the Western and Chinese models.

In order to test whether IDyOM is capable of simulating enculturation effects through statistical learning, IDyOM models were trained on corpora reflecting different musical cultures, simulating listeners from those cultures. A Western listener was simulated by training a model on a corpus of Western folk songs (the Western model) and a Chinese listener by training a model on a corpus of Chinese folk songs (the Chinese model). Each model was used to make both within‐culture and between‐culture predictions. For the within‐culture predictions (i.e., the Western model processing Western folk songs or the Chinese model processing Chinese folk songs), IDyOM was used to estimate the IC of every event in every composition in the corpus (using 10‐fold cross‐validation^147^ to create training and test sets from the same corpus). For between‐culture predictions, IDyOM was first trained on the within‐culture corpus (e.g., the Western corpus for the Western model) and then used to estimate the IC of every note in every composition in the other corpus representing the comparison culture (e.g., the Chinese corpus for the Western model). IDyOM was configured to use only its LTM trained on the appropriate corpus; the short‐term model was not used. In all cases, IC was averaged across notes, yielding a mean IC value representing the unpredictability of each composition for each model. The results are shown in Figure 4 and Table 1.

**Table 1:** IDyOM simulations of cultural distance between the Chinese and Western corpora (Fig. 4)

	Western example (deut1445)			Chinese example (han0418)			Overall	
	Western model IC	Chinese model IC	Cultural distance	Western model IC	Chinese model IC	Cultural distance	Accuracy	Cultural distance
Pitch	2.44	6.53	2.89	4.77	2.36	1.70	97.91	0.62
Onset	1.49	2.86	0.97	4.51	3.11	0.99	84.27	0.15
Pitch and onset	3.93	9.39	3.86	9.27	5.48	2.69	98.52	0.77

note: Results are shown for IDyOM models configured to predict pitch only (using an attribute linking pitch interval with scale degree, pi ⊗ sd, see Fig. 1), onset only (using the attribute ioi‐ratio), and both pitch and onset. Overall accuracy and cultural distance are shown as well as results for a Western and a Chinese piece with high cultural distance (Fig. 4) including the information content (IC) for the Western and Chinese models (trained on the Western and Chinese corpora, respectively) and cultural distance.

For the comparison between cultures (Western versus Chinese), the data are plotted in Figure 4 for each composition in the two corresponding corpora: IC for one model is plotted on the abscissa, while IC for the second model is plotted on the ordinate. The line of equality (*x* = *y*) indicates equivalence between the two models: compositions lying on this line are equally predictable for each model and do not distinguish the two cultures; in other words, they should be equally familiar and predictable to listeners enculturated in either musical style. Positions near the origin represent compositions that are predictable within both cultures, while positions far from the origin represent compositions that are unpredictable within both cultures. Positions farther away from the line of equality represent compositions that are predictable for the simulated model of one culture but unpredictable for the simulated model of the other culture. Distance from the line of equality, therefore, provides a quantitative measure of cultural distance^138^ based on information‐theoretic modeling of enculturation in musical styles. Figure 4A illustrates how cultural distance is computed for a comparison between IDyOM models trained on Western and Chinese corpora and, by rotating the data points through 45°, Figure 4B shows the same data with cultural distance on the ordinate and culture‐neutral complexity on the abscissa. In this example, IDyOM correctly classifies 98.52% of the folk songs by culture (Chinese versus Western). Moreover, classification accuracy and cultural distance are greater for IDyOM models configured to predict both pitch and time than models configured to predict pitch or time in isolation (Table 1), suggesting both that a combination of temporal and pitch regularities distinguishes the styles and that IDyOM is capable of learning such distinctive regularities in pitch and timing.

This approach provides a formal, computational model of enculturation, which guides the proposition of hypotheses about cultural familiarity and processing fluency. For example, referring to the examples shown in Figure 4, stimuli with strongly positive cultural distance should prove culturally familiar and easy to process for Western listeners but culturally unfamiliar and difficult to process for Chinese listeners and vice versa for stimuli with strongly negative cultural distance.

Van der Weij *et al*. developed empirical simulations of the effects of enculturation on metrical inference, using the computational model of metrical inference described above.^132^ A Western model trained on 1136 German folk songs is compared with a Chinese model trained on 1136 Chinese folk songs (all stimuli taken from the Essen Folk Song Collection^135^, ^136^, ^137^). When tested on 200 unseen folk songs from each culture, the Western model shows greater IC (prediction error) for Chinese music (1.72 bits per symbol) than for German music (1.34), while the Chinese model shows greater prediction error for the German music (1.70) than the Chinese music (1.49). Furthermore, the Western model also shows better meter‐finding performance for German music (73% correct) than Chinese music (72%), while the Chinese model performs better on Chinese music (75%) than German music (47%).

These simulations demonstrate that IDyOM provides a plausible computational model of enculturation effects through statistical learning, though further empirical studies are required to fully corroborate SLH.

## Conclusions

I have proposed two hypotheses about the psychological processes underlying enculturation in musical styles: (1) that probabilistic prediction is a foundational process in music perception underpinning other psychological processes (PPH) and (2) that statistical learning is the mechanism by which listeners acquire probabilistic models of musical styles (SLH). A review of the empirical evidence demonstrates that many different aspects of music perception—expectation, emotional response, recognition memory, phrase boundary perception, perceptual similarity, and, potentially, meter perception—can be simulated in terms of a single underlying process of probabilistic prediction, implemented in IDyOM. While these results are consistent with the SLH, since an IDyOM model trained on Western music accurately simulates Western listeners across a range of tasks, they do not provide causal evidence for the SLH. However, the results of a recognition memory study^108^ show that memory performance is causally related to dynamic statistical learning of an artificial musical system. Finally, I presented data from computational simulations suggesting that statistical learning can plausibly predict causal effects of differential cultural exposure to musical styles on perception, providing a formal, quantitative model of cultural distance.^138^

Therefore, there are increasingly valid empirical and theoretical grounds to propose probabilistic prediction based on statistical learning as a foundational psychological process in a general theory of music perception. However, several areas remain open for future research. The results reviewed in this paper have been obtained for discrete, symbolic representations of melodic musical styles. To generalize the approach to a wider variety of musical styles, the representational capacity of IDyOM must be expanded to polyphonic music^61^ but also to musical cultures that have no written tradition, where the distinction between composition and performance is blurred or nonexistent, or where music is inextricably combined with other modes of communication.^148^, ^149^ Doing so would open up the approach to a much broader range of musical cultures and traditions while also introducing significant computational challenges in modeling statistical learning and probabilistic prediction. It is also important to understand in more detail how musical training (active and explicit) and musical exposure (passive and implicit) exert a combined influence in musical enculturation.^150^, ^151^ Questions also arise over the effects of exposure to more than one musical style during enculturation. Further research is required to examine whether IDyOM's statistical learning mechanism distinguishes the styles sufficiently to account for such cases of bimusicalism or whether separate IDyOM models simulate bimusical listeners more accurately.^152^, ^153^ Finally, there is a fast‐growing body of neuroscientific research on predictive processing in music,^80^, ^88^, ^89^, ^91^, ^92^, ^96^, ^154^, ^155^ and further progress in understanding the neural processes underlying the SLH and the PPH in musical enculturation will benefit significantly from closely coordinated combination of empirical neuroimaging with computational modeling of the underlying mechanisms as outlined in this paper.

## Competing interests

The author declares no competing interests.
